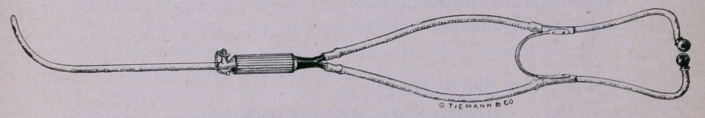# A New Stone Searcher

**Published:** 1896-08

**Authors:** Lucien Lofton

**Affiliations:** 306, 308 Equitable Building; Atlanta, Ga.


For the Texas Medical Journal.

### A NEW STONE SEARCHER.

BY LUCIEN LOFTON, M. D., ATLANTA, GA.

SO FAR as the literature upon genito urinary surgery is concerned, no one has up to the present time presented to the medical profession a stone searcher like the one herewith shown. I hope my instrument will be kindly received by practitioners in medicine and surgery.

As shown in the above cut, the detection of a stone depends upon direct transmission from the foreign body to the ear. The instrument is very sensitive, and a positive diagnosis may be reached, as a rule, by one sitting. The searcher is simplicity itself; and any surgeon who may possess an average degree of auditory sense, can in an instant detect the object searched for.

I claim this stone searcher will prove to the bladder what the stethoscope has to the chest. It is needless for me to remind the profession of the trouble one often experiences in trying to find foreign bodies in the bladder by the old method. Not reflecting in any sense upon other searchers, I only am glad that I am able to give to genito urinary surgery an instrument which will, in my opinion, simplify matters completely.

It is advised that due notice is taken upon inserting or withdrawing the sound, on account of stones being sometimes imbedded in the urethral tract. Should a vesical calculus be covered with bladder debris, contact with the searcher will reveal the identity of the body touched; likewise when a stone is encysted or lies protected by folds of vesical mucous membranes.

Strictures of sufficient size to pass the searcher, will give a soft grating noise which is peculiar, and when heard once will always be recognized.

Two sizes of the searcher is made; one for adults, and one for infants and children. The regulation Thompson curve is made beside the one I have just suggested. The bullet probe and the œsophogeal sound may be used upon the same principle.

I herewith give a short description of the invention:

As shown by the cut, which has been kindly furnished by Messrs. Tiemann & Co., of New York, who manufacture the instrument, the seacher can be readily attached to any of the newer forms of stethoscopes. The searcher consists of a hollow sound having two eyes or openings in the curve, and an outlet with the plug near the handle; a hollow corrugated metal handle terminating in a solid screw receiving the hard rubber metal lined connecting piece, to the two branches of which the stethoscope are attached.

306, 308 Equitable Building.

## Figures and Tables

**Figure f1:**